# Erratum

**DOI:** 10.4269/ajtmh.944err

**Published:** 2016-04-06

In *Am J Trop Med Hyg 92:* 1038–1044 by Schoeps and others, there is a small layout error in Table 2, which may cause misinterpretation. The variable “year of birth” is indented, so it appears to be one category of the above variable “twin birth”. “Year of birth” is a separate variable that is not part of “twin birth”. The journal regrets the error.

